# Nitazoxanide is active against *Mycobacterium leprae*

**DOI:** 10.1371/journal.pone.0184107

**Published:** 2017-08-29

**Authors:** Mai Ann Bailey, Hana Na, Malcolm S. Duthie, Thomas P. Gillis, Ramanuj Lahiri, Tanya Parish

**Affiliations:** 1 Infectious Disease Research Institute, Seattle, Washington, United States of America; 2 Department of Health and Human Services, Health Resources and Services Administration, Healthcare Systems Bureau, National Hansen’s Disease Program, Baton Rouge, Louisiana, United States of America; Indian Institute of Technology Delhi, INDIA

## Abstract

Nitazoxanide (NTZ) is an anti-parasitic drug that also has activity against bacteria, including *Mycobacterium tuberculosis*. Our data using both radiorespirometry and live-dead staining *in vitro* demonstrate that NTZ similarly has bactericidal against *M*. *leprae*. Further, gavage of *M*. *leprae*-infected mice with NTZ at 25mg/kg provided anti-mycobacterial activity equivalent to rifampicin (RIF) at 10 mg/kg. This suggests that NTZ could be considered for leprosy treatment.

## Introduction

Leprosy, the complex spectral disease caused by *Mycobacterium leprae* infection, remains a global health concern despite sustained efforts to eliminate it over the last 3 decades. Depending upon their disease presentation, leprosy patients are currently treated for 6–12 months with multidrug therapy (MDT), an antibiotic cocktail consisting of clofazimine, dapsone and rifampicin (RIF) [1]. Many patients experience side effects and toxicity attributable to each drug or simply become weary with the length of treatment [2, 3]. Clofazimine has a weakly bactericidal action against *M*. *leprae* and, because it often causes discoloring of the skin, patients commonly withdraw clofazimine from their treatments. Although it has anti-inflammatory and immunomodulatory effects via blockade of myeloperoxidase, the precise antibacterial mechanism of action of dapsone is not known. Dapsone was originally used as a monotherapy and resistance is now relatively widespread. RIF is the only drug incorporated in the current MDT regimen that is strongly bactericidal for *M*. *leprae* [4, 5], and many patients may unintentionally be receiving RIF monotherapy, a situation conducive for the emergence of RIF-resistant *M*. *leprae*. While resistance levels are not currently causing alarm, the widespread emergence of RIF-resistance has the potential to undermine the efforts of the WHO-MDT campaign [6–10]. A Sentinel Surveillance Network has been implemented to proactively monitor the situation [11], and the identification and validation of additional and alternative bactericidal agents that can treat leprosy appears prudent.

Nitazoxanide (NTZ: 2-acetyloxy-N-(5-nitro-2-thiazolyl)benzamide) is a broad-spectrum anti-parasitic drug used against a wide variety of parasites, including both protozoans and helminths [12–14], and it has been proposed that, acting as PDI inhibitor, NTZ could be added as a new and potent chemotherapeutic strategy against several cancers. Of particular note, NTZ has also demonstrated activity against *Mycobacterium tuberculosis* [15–17]. Given the familial relationship between *M*. *tuberculosis* and *M*. *leprae* we hypothesized that NTZ would also be efficacious against *M*. *leprae*.

Strategies to identify potential anti-leprosy drugs are complicated because culture systems that permit axenic growth of *M*. *leprae* are not available and antibacterial activity cannot therefore be monitored by the classic *in vitro* methods. Although recent advances have indicated that *M*. *leprae* can survive in medium for a limited length of time, drugs with anti-leprosy potential have classically been evaluated in the mouse footpad model, a protracted *in vivo* testing system that requires access to live *M*. *leprae* and large numbers of animals [18]. The short-term survival of *M*. *leprae* in culture does, however, provide the opportunity to conduct at least modified screening in a low throughput fashion to reduce the quantity of *in vivo* screening required. Thus, in this report, we used short-term *in vitro* and longer term *in vivo* screening methods to evaluate the potential of NTZ as a drug candidate for leprosy.

## Materials and methods

### *M*. *leprae* harvest

Live *M*. *leprae* bacilli (Thai-53 strain) were extracted from the footpads of *nu*/*nu* mice at National Hansen’s Disease Programs under NIH Contract IAA-2646 and either used internally or shipped overnight on ice to IDRI. The initial viability of all *M*. *leprae* used in these studies was greater than 80% as assessed by staining and radiorespirometry [19].

### Metabolic activity in axenic culture

Respiration was monitored over 14 days by evaluating the oxidation of ^14^C-palmitic acid to ^14^CO_2_ by radiorespirometry [20]. Briefly, 1 x 10^7^ *M*. *leprae* were suspended in 4 mL of acidified Middlebrook 7H12 BACTEC PZA medium (Becton Dickinson) in a 5 mL glass vial with loosened cap and placed into a wide mouth liquid scintillation vial lined with filter paper impregnated with NaOH, 2,5-diphenyloxazole (Sigma) and Concentrate I (Kodak). Results were calculated as counts per minute (cpm).

### Determination of bacterial viability

Live-dead staining procedures were adapted to monitor *M*. *leprae* viability, as assessed by membrane integrity, over time. Briefly, *M*. *leprae* were inoculated in 3 mL Luria-Bertani (LB) broth plus 0.05% w/v Tween to 1 x 10^6^ cells/mL. Cultures were then treated with Nitazoxanide (NTZ: 2-acetyloxy-N-(5-nitro-2-thiazolyl)benzamide) or Rifampicin (RIF; 3-(4-Methylpiperazinyliminomethyl)rifamycin) (Sigma Aldrich) and incubated at 33°C with 5% CO_2_. Samples were removed after various periods of time, the bacteria harvested by centrifugation and resuspended in 1 mL sterile distilled water. Bacteria were stained with the LIVE/DEAD® Baclight Bacterial Viability Kit (Invitrogen) with 5 μM SYTO 9 and 30 μM propidium iodide at room temperature in the dark for 10 min and washed twice with distilled water. The bacterial pellet was resuspended in 5% glycerol in saline, 5 μl of the suspension was spread onto a glass slide. Three representative sample frames were captured by fluorescent microscopy using excitation/ emission (Ex/Em) of 480/500 nm for SYTO 9 and 490/536 nm for propidium iodide using a Nikon fluorescence microscope. Images were converted to binary versions, segmented using iterative watershedding, and particle images were analyzed for counts of the red and green bacilli, indicating dead and live bacteria, respectively. The percent of viable *M*. *leprae* under each culture condition was determined.

### Treatment of *M*. *leprae-*infected mice

To evaluate *in vivo* activity, female C57BL/6 mice (Charles River) were inoculated with 1 x 10^4^ live *M*. *leprae* by injection in both hind foot pads. After 12 weeks, daily gavage was initiated and maintained to provide a total of 20 administrations per mouse over four weeks. All animal procedures were conducted under a scientific protocol reviewed and approved by the the National Hansen's Disease Program Institutional Care and Use Committee (IACUC) (Assurance #A3032-01). After 20 weeks (i.e., 4 weeks after the final gavage), mice were sacrificed and the feet disinfected with 70% ethanol and Betadine to allow the skin to be removed and the foot pad tissue to be excised. Each foot pad tissue was stored in 70% ethanol at −20°C until it was processed for DNA purification. Molecular enumeration of *M*. *leprae* was determined by using the purified DNA fraction from each specimen and real time PCR technology using primers and a probe for a common region of the RLEP family of dispersed repeats in *M*. *leprae*, as previously described [21]. PCR and data analyses were performed on a 7300 Real Time PCR System (Life Technologies), with *M*. *leprae* burdens calculated by extrapolating into a standard curve generated by preparing 4-fold serial dilutions of a known number of *M*. *leprae*.

### Statistics

*p*-values were determined using the Mann-Whitney rank sum test.

## Results

### NTZ is bactericidal against *M*. *leprae in vitro*

**Fig 1:**
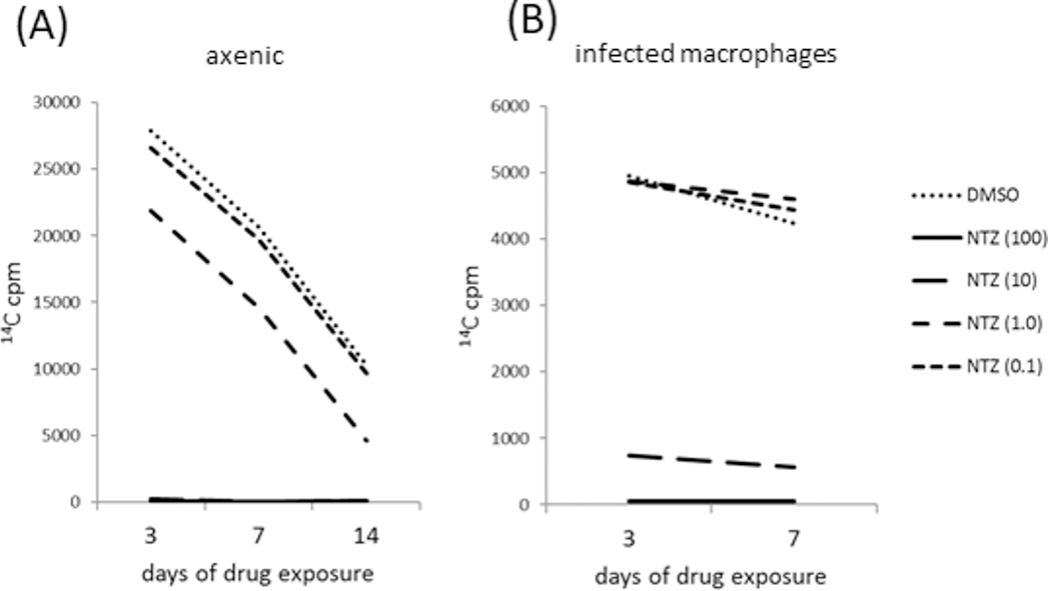
Metabolic activity of *M*. *leprae* treated with RIF and NTZ. *M*. *leprae* were cultured either (A) axenically or (B) following infection of macrophages. Metabolic activity of *M*. *leprae* was measured using radiorespirometry during 3–14 days of exposure to 2 μg /mL RIF or 0.1–100 μg/mL NTZ. Untreated control contained DMSO only. Results are representative of four experiments.

As an initial assessment of any potential activity of NTZ against *M*. *leprae*, bacterial cultures were inoculated with varying concentrations of drug and respiration measured over time. No ^14^C was incorporated in the cultures inoculated with the higher doses of NTZ indicating that respiration was completely inhibited at 100 μg/mL NTZ as early as day 3 of culture (Fig 1, and data not shown). The effect of NTZ was dose-dependent, as inoculation with 10 μg/mL NTZ partially reduced, and lower concentrations of NTZ (0.1 to 1 μg/mL) did not alter, the quantity of ^14^C incorporated from that observed in *M*. *leprae* cultures inoculated with DMSO (vehicle control). These data indicate that NTZ can inhibit *M*. *leprae* respiration in a dose-dependent manner, suggesting an inhibitory effect of NTZ on *M*. *leprae*.

**Fig 2:**
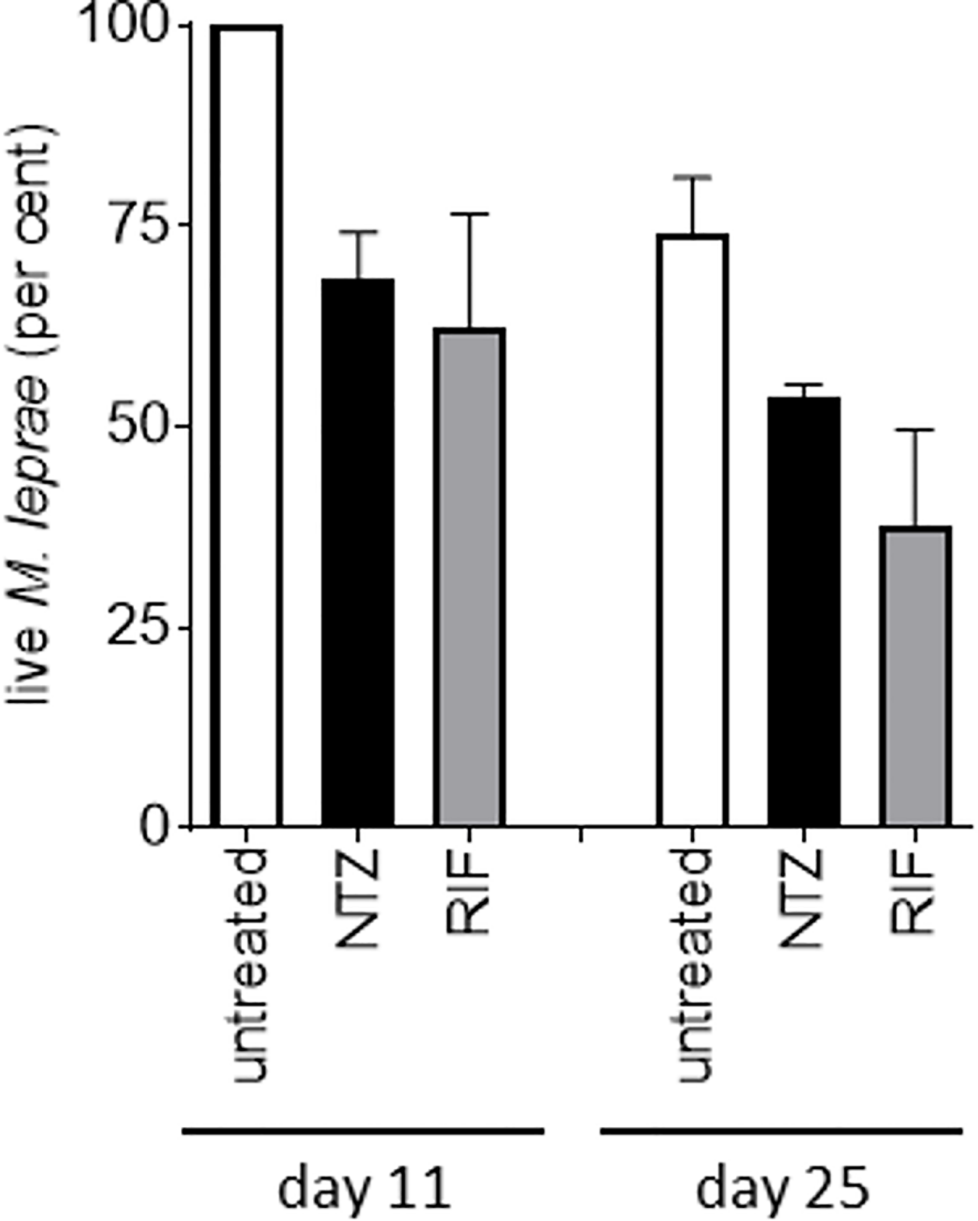
Bactericidal kinetics for RIF and NTZ against *M*. *leprae* in axenic medium. *M*. *leprae* was incubated in medium plus 2 μg/mL rifampicin (RIF) or 100 μg/mL nitazoxanide (NTZ). Viable bacteria were determined after 11 and 25 days using live-dead staining (SYTO 9/propidium iodide). Untreated control contained DMSO only. Results are shown as mean and standard deviation of 3 independent samples.

To further examine the anti-*M*. *leprae* activity of NTZ, we maintained *M*. *leprae* in axenic culture for extended periods of time and assessed their viability under antibiotic pressure. Under the control, untreated conditions the proportion of live *M*. *leprae* remained stable over 11 days and was slightly decreased after 25 days (Fig 2). In contrast, incubation in the presence of either NTZ or RIF resulted in a significant decrease in the proportion of live bacteria over time (Fig 2). These data indicate that NTZ exhibits bactericidal activity against *M*. *leprae*.

### Inhibition of *M*. *leprae* by NTZ treatment

**Fig 3:**
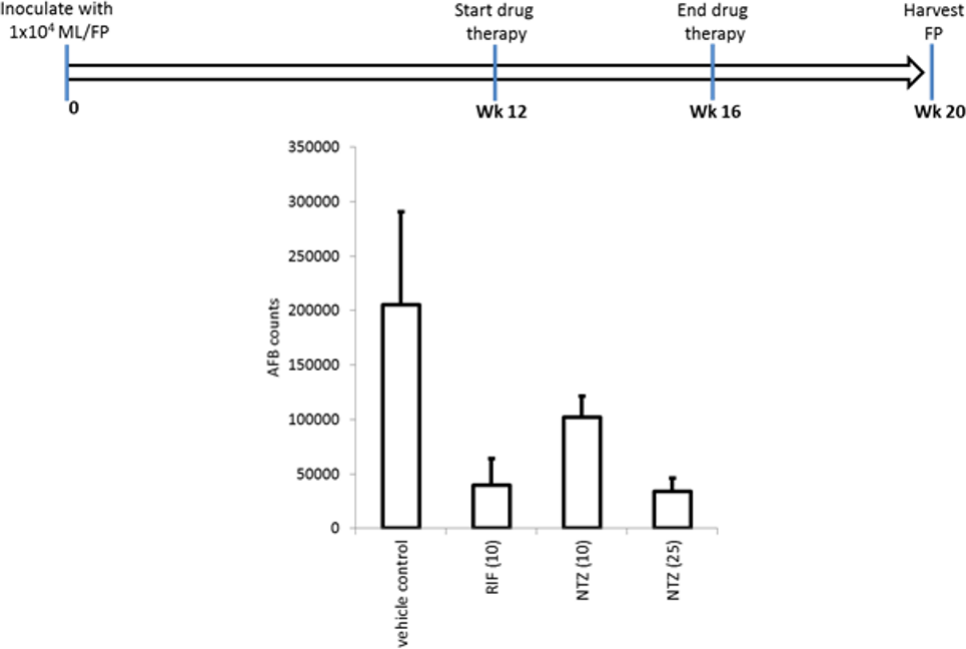
NTZ demonstrates *in vivo* activity against *M*. *leprae*. Female, 4–6 weeks old C57BL/6 mice were inoculated with 1 x 10^4^ live *M*. *leprae* in both hind foot pads. After 12 weeks, mice were divided into 10 mice per group and daily gavage with drugs was started (5 days per week for four consecutive weeks). Four treatment groups were established: vehicle control; RIF 10mg/kg; NTZ 10mg/kg; and NTZ 25mg/kg. At 20 weeks (4 weeks after the last gavage), mice were sacrificed and foot pads harvested to determine *M*. *leprae* burden by RLEP (AFB) count by TaqMan. Mean and SD *M*. *leprae* burdens are shown. *** = *p*-value < 0.001 versus vehicle control or between the groups indicated by the horizontal bar.

To evaluate if NTZ was also effective *in vivo* in limiting *M*. *leprae*, mice were infected in the feet and the infection allowed to establish for 3 months before the initiation of daily drug treatment (Fig 3A). As expected, the *M*. *leprae* burdens observed in mice treated with 10mg/kg RIF were significantly reduced from those observed in mice treated with the vehicle alone (Fig 3B). A comparable reduction was achieved by treating mice with 25mg/kg NTZ, while the partial reduction observed in mice treated with 10mg/kg NTZ indicated a dose-dependency of NTZ treatment. Together, our data demonstrate that NTZ has activity against *M*. *leprae* and suggest that it could be considered as an alternate drug for the treatment of leprosy.

## Discussion

The current treatment regimen for leprosy, MDT, appears to be highly effective with the marked reduction in the incidence of leprosy have been attributed to its widespread dissemination and use. Although relapse rates after completing MDT are generally low (~1%), unacceptably high rates have been reported in some areas [3, 22–25] and resistance to and/or non-compliance with some of its components pressures its efficacy. Multidrug-resistant strains of *M*. *leprae* have been induced under laboratory conditions and are occasionally observed in patients [10, 26–30] The wider emergence of resistance, in particular to RIF, would be a dramatic setback to control efforts. It is likely that the addition of another drug to complement the bactericidal activity of RIF would be beneficial. As alternatives to MDT for the treatment of leprosy, moxifloxacin/pefloxacin/ofloxacin, minocycline and clarithromycin have all demonstrated greater activity than both dapsone and clofazimine in clinical trials. Actual application of these has been largely confined to the use of single dose rifampicin, ofloxacin and minocycline (ROM) for single lesion PB leprosy patients in trials [31–38]. It is noteworthy, however, that ofloxacin resistance has been observed in at least two relapses [7, 11, 31, 39–42]. Gatifloxacin, linezolid and moxifloxacin are all licensed to treat several bacterial infections and anti-mycobacterial activity has been demonstrated in TB models. These drugs have demonstrated activity against replicating *M*. *leprae* in the mouse footpad model have been undertaken in the mouse footpad model. [43–45] Thus, experimental evaluations of emerging anti-mycobacterials that are being driven by TB research provides an important transition to inform their potential, or lack thereof, for treating leprosy. Our data identify NTZ as a potential anti-leprosy drug and, in the mouse model, indicated that repeated treatment with a 25 mg/kg dose of NTZ had bactericidal activity against *M*. *leprae* that was equivalent to 10 mg/kg RIF. Knowing that NTZ is available as an alternative drug that can readily be deployed provides some comfort for current efforts to sustain leprosy control.

It is unclear how NTZ exerts its activity against *M*. *leprae*. NTZ can induce autophagy in mammalian cells and this could be an important drug-induced control pathway given that it appears that *M*. *leprae* can inhibit autophagic machinery as part of its immune evasion strategy [46–48]. The fact that NTZ can kill replicating and nonreplicating *M*. *tuberculosis* is surprising given that Mtb does not possess a homologue for the putative bacterial target, pyruvate ferredoxin oxidoreductase (PFOR) [49]. Interestingly, attempts to generate NTZ-resistant *M*. *tuberculosis* colonies were unsuccessful and suggest that multiple mechanisms of action may contribute [50]. Among the potential mechanisms, it has been demonstrated that NTZ treatment disrupts both the membrane potential and intrabacterial pH homeostasis of *M*. *tuberculosis* [16]. It is likely that these also occur for *M*. *leprae* and, indeed, our live/ dead fluorescent staining supports the notion that membrane integrity is negatively impacted by NTZ treatment.

NTZ has now been licensed in the United States for the treatment of intestinal infections caused by *Cryptosporidium parvum* [51–53]. In addition, it can be used as an antiviral agent and it is therefore also undergoing clinical development for treatment of influenza and other viral respiratory infections [13]. At present, NTZ is widely commercialized and used as a broad-spectrum antiparasitic agent in several leprosy endemic regions, including throughout Latin America and the Indian subcontinent. Given this range of use it is possible that leprosy patients, or *M*. *leprae*-infected individuals who are not displaying disease symptoms, may receive NTZ for the treatment of other conditions. Together with our data, this raises the possibility that the *M*. *leprae* infection would therefore also be inadvertently treated with NTZ. Taken together, this information and our data suggest that NTZ could be considered as a supplement to the drug arsenal for the treatment of leprosy.
